# Time-Based Capnography to Diagnose Airway Obstruction During Lung Lobectomy in a Dog

**DOI:** 10.3390/ani15233368

**Published:** 2025-11-21

**Authors:** Toshitsugu Ishihara, Li-Jen Chang

**Affiliations:** Department of Small Animal Clinical Sciences, Virginia-Maryland College of Veterinary Medicine, Blacksburg, VA 24061, USA; ljchang@vt.edu

**Keywords:** capnography, airway obstruction, anesthesia, lung lobectomy, dog

## Abstract

A 10-year-old, 7.0 kg, female spayed mixed-breed dog presented for a second opinion regarding lobectomy for a lung mass. Mechanical ventilation was initiated due to hypercapnia (ETCO_2_ of 61 mmHg) with PIP of 8 cmH_2_O. The PIP was gradually increased to 16 cmH_2_O. Six minutes after increasing PIP to 16 cmH_2_O, ETCO_2_ suddenly dropped to 0 mmHg and the capnography waveform was lost. The endotracheal (ET) tube connection to the ventilator circuit was intact, and no visible abnormalities were noted on the ET tube. Although the ETCO_2_ sampling line and water trap, and the monitor were replaced, the ETCO_2_ remained at 0 mmHg. Meanwhile, suspecting obstruction within the ET tube or the airway, extubation was performed. A large blood clot in the ET tube was noticed. Reintubation was performed using a new ET tube and mechanical ventilation was restarted with PIP set to 10 cmH_2_O. The ETCO_2_ showed a reading of 55 mmHg. Five minutes after reintubation, the ETCO_2_ waveform disappeared again. The inside of ET tube was suctioned, and a blood clot was again identified. Clinicians should be aware of these risks when performing lung lobectomy without one lung ventilation.

## Simple Summary

Capnography is a technique for continuously and non-invasively monitoring end-tidal carbon dioxide and inspired carbon dioxide as a number and waveform. Time-based capnography is useful not only for evaluating the carbon dioxide partial pressure in respiratory gases and ventilation status, but also for differential diagnosis of hypoxemia, assessing the integrity of endotracheal tubes and breathing circuits, and evaluating pulmonary perfusion. The American College of Veterinary Anesthesia and Analgesia Small Animal Anesthesia and Sedation Monitoring Guidelines 2025 recommends continuous patient monitoring using time-based capnography as one of the minimum requirements. This case report describes a sudden loss of end-tidal carbon dioxide during general anesthesia in a dog during lung lobectomy, along with differential diagnoses and management. Although the hemorrhage was possibly caused by the increased peak inspiratory pressure, a definitive conclusion could not be reached. Even though one-lung ventilation is considered to resolve this issue, since one-lung ventilation may not be available at all facilities and may be difficult to use depending on the size of patients, veterinarians should recognize that performing a lung lobectomy without one-lung ventilation carries the potential risk of airway obstruction due to pulmonary hemorrhage. Continuous monitoring of end-tidal carbon dioxide is therefore imperative.

## 1. Introduction

Capnography is a technique for continuously and non-invasively monitoring CO_2_ partial pressure during the respiratory cycle and a comprehensive measurement and display of CO_2_, including end-tidal CO_2_ (ETCO_2_) and inspired CO_2_ as a number and waveform [1]. Time-based capnography refers to the expired volume of CO_2_ over time. Exhaled CO_2_ can be sampled via either mainstream or side-stream expiratory flow. In the mainstream approach, the infrared light source and sensor are placed within the main airflow tube to directly sample exhaled gases during expiration [2]. In the side-stream approach, gas is continuously aspirated from the airway through a sampling line that is placed between the endotracheal tube and the Y-piece of the circuit [2]. Time-based capnography is useful not only for evaluating the partial pressure of carbon dioxide (PaCO_2_) in respiratory gases and ventilation status, but also for differential diagnosis of hypoxemia, assessing the integrity of endotracheal tubes and breathing circuits, and evaluating pulmonary perfusion [3,4,5]. The American College of Veterinary Anesthesia and Analgesia Small Animal Anesthesia and Sedation Monitoring Guidelines 2025 recommends continuous patient monitoring using time-based capnography as one of the minimum requirements [6].

Compared to humans, primary lung cancer in dogs is relatively rare [7,8], accounting for less than 1% of canine tumors [9,10]. However, when they develop, surgical resection with or without chemotherapy is the primary treatment option [11]. When the thoracic cavity is opened for surgical resection, positive pressure ventilation is performed because achieving adequate ventilation becomes difficult [12,13].

This case report describes a sudden loss of ETCO_2_ was encountered during lung lobectomy under positive pressure ventilation for pulmonary tumor resection, including the clinical presentation, management, and outcome.

## 2. Case Presentation

A 10-year-old, 7.0 kg (body condition score 5 out of 9) spayed female mixed-breed dog presented to a primary hospital with a chief complaint of a persistent cough lasting two months and thoracic radiographs revealed a mass effect of soft tissue density in the left caudal lung lobe. Other thoracic structures were within normal limits. Thoracic ultrasonography revealed a heterogeneous and hypoechoic mass in the right caudal lung lobe. Percutaneous transthoracic fine-needle aspirates were not performed. CT scans revealed a solitary mass of 25 mm × 21 mm × 18 mm in the right caudal lung lobe and no obvious evidence of metastasis was found. Five days after the CT scan in the primary hospital, the patient was presented to the Auburn University Small Animal Teaching Hospital for a second opinion regarding surgical treatment.

The dog had not received any medications. The patient was coughing several times a day at home but had no hemoptysis and was energetic with a good appetite. Physical examination showed heart rate (HR) of 90 beats per minute, respiratory rate (RR) of 30 breaths per minute and a rectal temperature (T) of 38.4 °C. No murmurs or arrhythmias were auscultated. Normal lung sounds were auscultated, and no cough was noted at the hospital. The oral mucous membranes were pink and moist. Capillary refill time was less than 2 s. After the consultation, the owner agreed to the second opinion and lung lobectomy. Additional CT scan was not planned. Blood test results were unremarkable. The dog was classified as having an American Society of Anesthesiologists risk classification of 3.

On the next day of consultation, lung lobectomy was performed. Maropitant (1 mg/kg; Cerenia, Pfizer Animal Health, New York, NY, USA) was administered subcutaneously (SC) 55 min before intramuscular premedication. The dog was premedicated with dexmedetomidine (0.005 mg/kg; Dexdomitor, Zoetis, Kalamazoo, MI, USA) and methadone (0.5 mg/kg; Methadone hydrochloride, Wilmington, NC, USA) intramuscularly (IM). After sedation was confirmed, A 22-gauge, 1-inch catheter (Surflo I.V. Catheter, Terumo Medical, Somerset, NJ, USA) was placed into a cephalic vein, and the dog received 100% O_2_ (4 L/min) through a mask for 4 min before induction of general anesthesia with ketamine (2 mg/kg; ketamine hydrochloride, Covetrus North America, Dublin, OH, USA) and propofol (1.5 mg/kg; PropoFlo™-28, Zoetis Inc., Kalamazoo, MI, USA) intravenously (IV). The dog was intubated with a 6.5 mm internal-diameter endotracheal (ET) tube (High-volume, low-pressure (HVLP) Cuffed Oral/Nasal Endotracheal Tube with Murphy Eye, Medline Industries, LP, Northfield, IL, USA). The dog was then connected to a rebreathing circuit on an anesthesia machine (SurgiVet CDS 9000 Small Animal Anesthesia Machine, Smiths Medical, Waukesha, WI, USA) for delivery of 1.4% (vaporizer setting) isoflurane (Fluriso, VetOne, Boise, ID, USA) in 100% oxygen (0.5 to 3 L/min). The dog was monitored with electrocardiography, oxygen saturation (SpO_2_), ETCO_2_, end-tidal isoflurane concentration, HR, T, non-invasive blood pressure and invasive blood pressure at 5-min intervals during anesthesia (DRE Waveline Pro; DRE Medical Inc., Louisville, KY, USA). A doppler ultrasonic flow detector (Doppler flow detector Model 811-B; Parks Medical Electronics Inc., Aloha, OR, USA) was used for the continuous monitoring of the blood flow with the piezoelectric crystal placed over the palmar digital artery. Invasive blood pressure through a 22-gauge, 1-inch catheter placed in the dorsal pedal artery. The dog was provided thermal support using a forced air warming system (3M^TM^ Bair Hugger^TM^, Arizant Healthcare Inc., St. Paul, MN, USA) and external heat pad (HotDog^TM^ Patient Warming System, Augustine Temperature Management LLC, Eden, MN, USA). Intravenous fluid therapy was initiated with a crystalloid solution (5 mL/kg/hour; Vetivex Veterinary Lactated Ringer’s Injection, Dechra Veterinary Products, Overland Park, KS, USA). Additionally, ketamine 1 mg/kg/hour constant rate infusion (CRI) was started. Cefazoline IV (22 mg/kg mg/kg; MWI Animal Health, Boise, ID, USA) was administered every 90 min, and the dog was placed in the left lateral recumbency for clipping and scrubbing of the surgical area. Thirty-five minutes after anesthetic induction, intercostal nerve block was performed using a portable ultrasound device (Sonosite EDGE, SonoSite Inc., Bothell, WA, USA). 0.5% bupivacaine (2 mg/kg; Hospira Inc., Lake Forest, IL, USA) was administered at five sites between the fourth and eighth intercostal spaces. After the nerve block, the dog was transferred to an operating room and positioned in left lateral recumbency.

**Figure 1:**
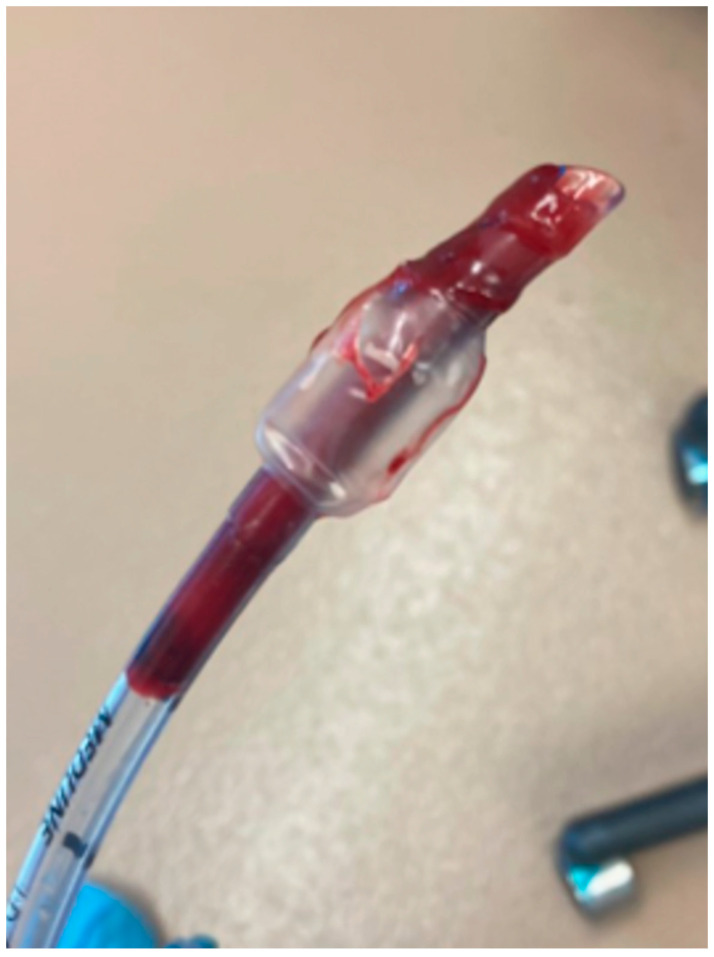
Extubation was performed after an abrupt loss of the ETCO_2_ waveform. A large blood clot was observed in the endotracheal tube.

In the operating room, mechanical ventilation (MV) (Model 2002 Anesthesia Ventilator; Hallowell EMC, Pittsfield, MA, USA) was initiated due to hypercapnia (ETCO_2_ of 61 mmHg) with setting of tidal volume of 12 mL/kg, RR of 8 breaths per minute, and peak inspiratory pressure (PIP) of 8 cmH_2_O. Five minutes after the MV was set, the ETCO_2_ remained at 52 mmHg. Therefore, PIP was increased to 10 cmH_2_O, resulting in ETCO_2_ of 44 mmHg. Lateral thoracotomy at the 6th right intercostal space was initiated 79 min after induction of anesthesia. Twenty minutes after the start of thoracotomy, ETCO_2_ was 55 mmHg and SpO_2_ was 100%. At this point, arterial blood gas was analyzed by i-STAT 1 Portable Clinical Analyzer (Abbott Laboratories, Abbott Park, IL, USA) with i-STAT CG8+ cartridges. The results showed partial pressure of oxygen in arterial blood (PaO_2_) of 440 mmHg and partial pressure of carbon dioxide in arterial blood (PaCO_2_) of 62 mmHg. The ventilator setting was then adjusted accordingly with PIP of 14 cmH_2_O and RR of 10 breaths per minute. ETCO_2_ reached 46 mmHg afterward. Additionally, Glycopyrrolate (0.01 mg/kg; Fresenius Kabi, Lake Zurich, IL, USA) was administered intravenously because the mean arterial pressure (MAP) was 60 mmHg, and HR was 48 beats per minute. Twelve minutes after administration of glycopyrrolate, MAP was 75 mmHg with HR 80 beats per minute, and ETCO_2_ was 54 mmHg. Because ETCO_2_ was relatively high again, PIP was set to 16 cmH_2_O, which lowered ETCO_2_ to 42 mmHg. Six minutes after setting PIP to 16 cmH_2_O, ETCO_2_ suddenly dropped to 0 mmHg and the waveform was lost. The SpO_2_ was 100% at the meantime, the endotracheal tube connection to the ventilator circuit was intact, and no visible abnormalities were noted on the ET tube. Additionally, the patient’s lungs were moving in synchronization with the rhythm of the mechanical ventilation as observed visually. A small amount of water condensation was found in the ETCO_2_ sampling line. The sampling line, ETCO_2_ water trap, and monitor were immediately replaced, but ETCO_2_ remained at 0 mmHg. Meanwhile, suspecting obstruction within the endotracheal tube or the airway, extubation of the ET tube was performed. A large blood clot within the ET tube was noticed (Figure 1). Reintubation was performed using a new endotracheal tube of the same size. Mechanical ventilation was restarted with PIP set to 10 cmH_2_O and RR set to 8 breath per minute. ETCO_2_ showed a reading of 55 mmHg. Five minutes after re-intubation, the ETCO_2_ waveform disappeared again. We cleaned the tube with vacuum suction and found a blood clot again. A thin, flexible tube was attached to the vacuum suction device and inserted into the tracheal tube to suction out the blood clot. After the suctioning, ETCO_2_ showed a reading of 55 mmHg and Blood gas analysis showed PaCO_2_ 61 mmHg and PaO_2_ 420 mmHg. After the intervention, ETCO_2_ remained between 55 and 57 mmHg, and SpO_2_ remained between 99 and 100% till the end of the procedure. After completion of the lung lobectomy, a chest tube was placed on the same side of the surgical side. Following administration of carprofen SC (4.4 mg/kg; Rimadyl, Zoetis, Kalamazoo, MI, USA) and acepromazine IV (0.01 mg/kg; VetOne, Boise, ID, USA), the ketamine CRI dose was reduced to 0.2 mg/kg/h, and the patient was transferred to the recovery room. The patient was placed in sternal recumbency, and general anesthesia was discontinued after evacuation of air from the chest tube. The patient was extubated 15 min after discontinuation of inhalant anesthesia. The duration of the surgical procedure was 1 h and 55 min. The total anesthesia time was 3 h 24 min. Throughout the procedure, the end-tidal isoflurane concentration was maintained between 1.1% and 1.2%, body temperature was maintained between 35.6 °C and 37.3 °C, the Electrocardiography was interpreted as regular with the HR between 47 and 88 beats per minute, and the MAP was maintained between 60 mmHg and 77 mmHg. Urine output was not measured. The total volume of crystalloid solution administered was 114 mL. After extubation, palpation around the incision site revealed no pain. Final TPR was T 37.2 °C, P 90, R 18. The patient was transferred to the ICU, and ketamine CRI (0.2 mg/kg/h) was continued.

On the following day, the patient was eating, drinking, urinating, and defecating normally. All physical examination findings were within normal limits. The drain was removed on the third day of the procedure, and the patient was discharged. The histopathology revealed the pulmonary mass to be a pulmonary adenocarcinoma.

## 3. Discussion

Capnography is a continuous measurement that provides both a graphic display and numeric values of CO_2_ throughout the respiratory cycle. Measurement of ETCO_2_ is a validated surrogate of PaCO_2_ in humans [14,15] and domestic animals [16,17]. ETCO_2_ underestimates PaCO_2_ by approximately 1–7 mmHg in small animals [18] because of the presence of alveolar dead space [19]. Hypercapnia refers to a state where the PaCO_2_ exceeds 45 mmHg [20]. Hypercapnia may cause respiratory acidosis, increased sympathetic tone, arrhythmias, suppression of cardiac contractility, and decreased systemic vascular resistance [21]. On the other hand, hypercapnia secondary to reduced alveolar ventilation is recognized in clinical practice as an element of protective ventilation [22]. Both clinical and experimental studies have demonstrated that therapeutic hypercapnia contributes to improving sepsis severity, ventilator-associated injury, and acute respiratory distress syndrome (ARDS) [23,24,25,26]. If permissive hypercapnia is used, PaCO_2_ < 80 mmHg, pH > 7.20; and PaO_2_ > 80 are suggested limits [27]. Furthermore, permissive/therapeutic hypercapnia, maintaining PaCO_2_ at 50–70 mmHg, may be beneficial in patients undergoing lobectomy with one-lung ventilation [22]. However, in this case, MV was initiated due to hypercapnia. The highest PIP we eventually reached was 16 cmH_2_O.

ETCO_2_ was lost 6 min after initiating MV at PIP 16 cmH_2_O. Common causes for absent ETCO_2_ levels and waveform include apnea, airway obstruction, cardiac arrest, malposition of endotracheal/tracheostomy tube, disconnection of the ventilator circuit, and equipment failure [1]. Examples of equipment failure include sampling line obstruction caused by condensation or secretions [28] and malfunction of the water trap.

In this case, we visually confirmed lung movement and inspected the ventilator circuit, the ET tube connection and the ET tube within visible range when ETCO_2_ was lost to rule out the aforementioned common causes of ETCO_2_ loss. However, since no abnormalities were found, we replaced the ETCO_2_ sampling line and the ETCO_2_ water trap with new ones and replaced the entire monitor with a different one. The ETCO_2_ remained absent, leading us to suspect airway obstruction within the endotracheal tube or airway. We extubated and confirmed a large blood clot adhering to the ET tube. The clot was presumed to result from the tumor, with two possible causes: First, it appeared 6 min after the increase in PIP, suggesting that this level of pressure to the lung ruptured the tumor and caused hemorrhage. Preoperative blood examination showed platelet count, prothrombin time, and activated partial thromboplastin time within normal ranges. No issues with coagulation status during the surgery were observed in other parts of the body.

Examples of diseases inducing hemoptysis include neoplasia, trauma, and pulmonary hypertension [29]. Hemoptysis associated with malignant tumors is common, accounting for approximately 25% of all hemoptysis cases, with about 20% of lung cancer patients experiencing hemoptysis in humans [30]. Therefore, lung tumors are susceptible to cause hemorrhage. This condition to cause hemorrhage, combined with the elevation of the PIP, may have triggered the airway obstruction.

Barotrauma is a potentially lethal complication associated with mechanical ventilation. Most of the patients with pneumothorax from MV have underlying lung diseases; pneumothorax is rare in intubated patients with normal lungs [31]. Airway pressure exceeding 50 cmH_2_O is associated with an increased risk of alveolar rupture during MV in humans [32]. In animal studies, there is evidence to conclude that lung overdistension rather than high airway pressure is the primary cause of alveolar and interstitial injury [33,34]. In dogs, a recruitment airway pressure of 15 cmH_2_O for 30 s is recommended to improve hypoventilation. This provides equivalent hypoventilation improvement to PIPs of 25 and 35 cmH_2_O while reducing the risk of hyperventilation and lung overstretching [35]. In another study in healthy dogs, a recruitment maneuver at 40 cmH_2_O for 20 s induced temporary improvement in lung aeration and gas exchange, and no side effects such as pneumothorax were reported [36]. Conditions associated with pneumothorax in mechanically ventilated patients include lung cancer, tuberculosis, bronchiectasis, asthma, pulmonary fibrosis, acute respiratory distress syndrome and other connective tissue and rheumatologic diseases [31]. Therefore, the PIP of 16 cmH_2_O we applied to the patient is not a problematic level in healthy dogs, but its interaction with the tumor may have caused the airway obstruction.

For lung lobectomy, one-lung ventilation is the most commonly used technique to maintain ventilation and oxygenation during the operation [22]. Indications for one-lung ventilation include cases with abscesses or pulmonary hemorrhage, and patients requiring lung lobectomy or thoracoscopic surgery in humans [37]. In our case, if one-lung ventilation had been used, the airway obstruction might have been prevented, although complications such as hypoxemia could still have occurred due to the reduced total lung capacity. However, to the best of the author’s knowledge, no clear indications for one-lung ventilation have been established in veterinary medicine. Additionally, one-lung ventilation is challenging in small animals and requires special equipment such as fluoroscopy [38].

## 4. Conclusions

This case report describes a sudden loss of ETCO_2_ during general anesthesia in a dog during lung lobectomy, along with differential diagnoses and management. Although the hemorrhage was possibly caused by the increased PIP, a definitive conclusion could not be reached. Furthermore, OLV is considered to resolve this issue. However, since OLV may not be available at all facilities, veterinarians should recognize that performing a lung lobectomy without OLV carries the potential risk of airway obstruction due to pulmonary hemorrhage. Continuous monitoring of ETCO_2_ is therefore imperative.

## Data Availability

The datasets used and/or analyzed during the current study are available from the corresponding author upon reasonable request.

## Abbreviations

The following abbreviations are used in this manuscript:

ETCO_2_	End-tidal CO_2_
PaCO_2_	Carbon dioxide partial pressure
PIP	Peak inspiratory pressure
ET	Endotracheal
HR	Heart rate
RR	Respiratory rate
T	Rectal temperature
SC	Subcutaneously
IM	Intramuscularly
IV	Intravenously
SpO_2_	Oxygen saturation
CRI	Constant rate infusion
MV	Mechanical ventilation
MAP	Mean arterial pressure
